# PatA Regulates Isoniazid Resistance by Mediating Mycolic Acid Synthesis and Controls Biofilm Formation by Affecting Lipid Synthesis in Mycobacteria

**DOI:** 10.1128/spectrum.00928-23

**Published:** 2023-05-22

**Authors:** Kun Wang, Yimin Deng, Xujie Cui, Mengli Chen, Yanzhe Ou, Danting Li, Minhao Guo, Weihui Li

**Affiliations:** a State Key Laboratory for Conservation and Utilization of Subtropical Agro-bioresources, College of Life Science and Technology, Guangxi University, Nanning, China; South China Sea Institute of Oceanology

**Keywords:** mycolic acid, INH resistance, lipid synthesis, biofilm formation, mycobacteria

## Abstract

Lipids are prominent components of the mycobacterial cell wall, and they play critical roles not only in maintaining biofilm formation but also in resisting environmental stress, including drug resistance. However, information regarding the mechanism mediating mycobacterial lipid synthesis remains limited. PatA is a membrane-associated acyltransferase and synthesizes phosphatidyl-*myo*-inositol mannosides (PIMs) in mycobacteria. Here, we found that PatA could regulate the synthesis of lipids (except mycolic acids) to maintain biofilm formation and environmental stress resistance in Mycolicibacterium smegmatis. Interestingly, the deletion of *patA* significantly enhanced isoniazid (INH) resistance in M. smegmatis, although it reduced bacterial biofilm formation. This might be due to the fact that the *patA* deletion promoted the synthesis of mycolic acids through an unknown synthesis pathway other than the reported fatty acid synthase (FAS) pathway, which could effectively counteract the inhibition by INH of mycolic acid synthesis in mycobacteria. Furthermore, the amino acid sequences and physiological functions of PatA were highly conserved in mycobacteria. Therefore, we found a mycolic acid synthesis pathway regulated by PatA in mycobacteria. In addition, PatA also affected biofilm formation and environmental stress resistance by regulating the synthesis of lipids (except mycolic acids) in mycobacteria.

**IMPORTANCE** Tuberculosis, caused by Mycobacterium tuberculosis, leads to a large number of human deaths every year. This is so serious, which is due mainly to the drug resistance of mycobacteria. INH kills M. tuberculosis by inhibiting the synthesis of mycolic acids, which are synthesized by the FAS pathway. However, whether there is another mycolic acid synthesis pathway is unknown. In this study, we found a PatA-mediated mycolic acid synthesis pathway that led to INH resistance of in *patA*-deleted mutant. In addition, we first report the regulatory effect of PatA on mycobacterial biofilm formation, which could affect the bacterial response to environmental stress. Our findings represent a new model for regulating biofilm formation by mycobacteria. More importantly, the discovery of the PatA-mediated mycolic acid synthesis pathway indicates that the study of mycobacterial lipids has entered a new stage, and the enzymes might be new targets of antituberculosis drugs.

## INTRODUCTION

Mycobacterium tuberculosis, one of the deadliest pathogens, has infected about one-quarter of the global population (1, 2). It was estimated that there were 10.6 million cases of tuberculosis (TB) and more than 1.6 million deaths due to this disease in the world in 2021 (3). The main reason for the high pathogenicity and lethality of tuberculosis is that the pathogenic bacteria have evolved antibiotic resistance due to their intricate cell wall structure (4). As the core part of the cell envelope, the mycobacterial cell wall is composed mainly of mycolic acid, peptidoglycan, and arabinogalactan through covalent bonds, and the outer layer of the cell wall is composed of free and noncovalently bound lipids (5).

Mycolic acids are the key components of the mycobacterial cell wall, and they affect bacterial virulence, permeability, and survival (6, 7). Therefore, the importance of mycolic acid for mycobacteria is self-evident. The biosynthetic pathway for mycolic acids includes only the fatty acid synthase I (FAS-I) and FAS-II pathways of the FAS system, and the FAS pathway has been extensively studied (8, 9). Exploring the synthetic pathway for mycolic acid is an extremely important and arduous project. Isoniazid (INH) was the first reported synthetic drug, and it is one of the most effective compounds for treating tuberculosis (10, 11). INH kills M. tuberculosis by inhibiting the synthesis of mycolic acids in the mycobacterial cell wall (12). Therefore, the enzymes involved in the synthesis of mycolic acids are ideal and important antituberculosis drug targets. However, in addition to the FAS pathway, whether there are other metabolic pathways to synthesize mycolic acids is unclear, and the involved enzymes remain to be explored.

The synthesis of lipid-related enzymes can affect the bacterial colony surface morphology and biofilm formation in mycobacteria (13). However, the related mechanism is unclear, the involved enzymes remain to be explored, and the specific components of lipids also need to be identified. Microbial biofilm formation is a heterogeneous aggregation, and the biofilm is composed mainly of polysaccharides, proteins, and DNA (14, 15). The matrix of the biofilm is a physical barrier that provides a protective ecological niche for the survival of bacteria, eventually leading to the emergence of a drug-tolerant phenotype (16). Many pathogenic microorganisms such as Pseudomonas aeruginosa, Mycobacterium abscessus, and Candida albicans can form biofilms to resist host immune responses, antibacterial reagents, and drugs (17–19). The failure of antimicrobial therapy is generally attributed to the high level of drug-resistant bacteria embedded in the biofilm. Even highly sensitive bacteria without antibiotic resistance will generate drug resistance when they are embedded in a biofilm (20). Therefore, the relationship among bacterial biofilm formation, its lipid composition, and stress resistance remains to be explored.

Lipids account for more than 60% of the mycobacterial cell wall. There are many genes for lipid synthesis, but only a few have been successfully identified in mycobacteria. As a membrane-associated acyltransferase, PatA is involved in the biosynthesis of phosphatidyl-*myo*-inositol mannosides (PIMs) in M. tuberculosis (21). However, in addition to synthesizing PIMs, whether PatA is involved in mediating the synthesis of mycolic acids and other lipids in mycobacteria is unclear, and the related mechanisms remain to be explored. In this study, we found that PatA positively regulated mycobacterial biofilm formation and environmental stress resistance by mediating the synthesis of lipids (except mycolic acids), while it negatively regulated mycobacterial INH resistance by inhibiting the synthesis of mycolic acids, which is unexpected. This study discovered a relationship between PatA and mycobacterial INH and a link between PatA and biofilm formation. Meanwhile, we found an unknown mycolic acid synthesis pathway and revealed the molecular mechanisms of lipid metabolism and their correlation with bacterial physiological phenotypes.

## RESULTS

### PatA affects colony surface morphology and biofilm formation in M. smegmatis.

**FIG 1:**
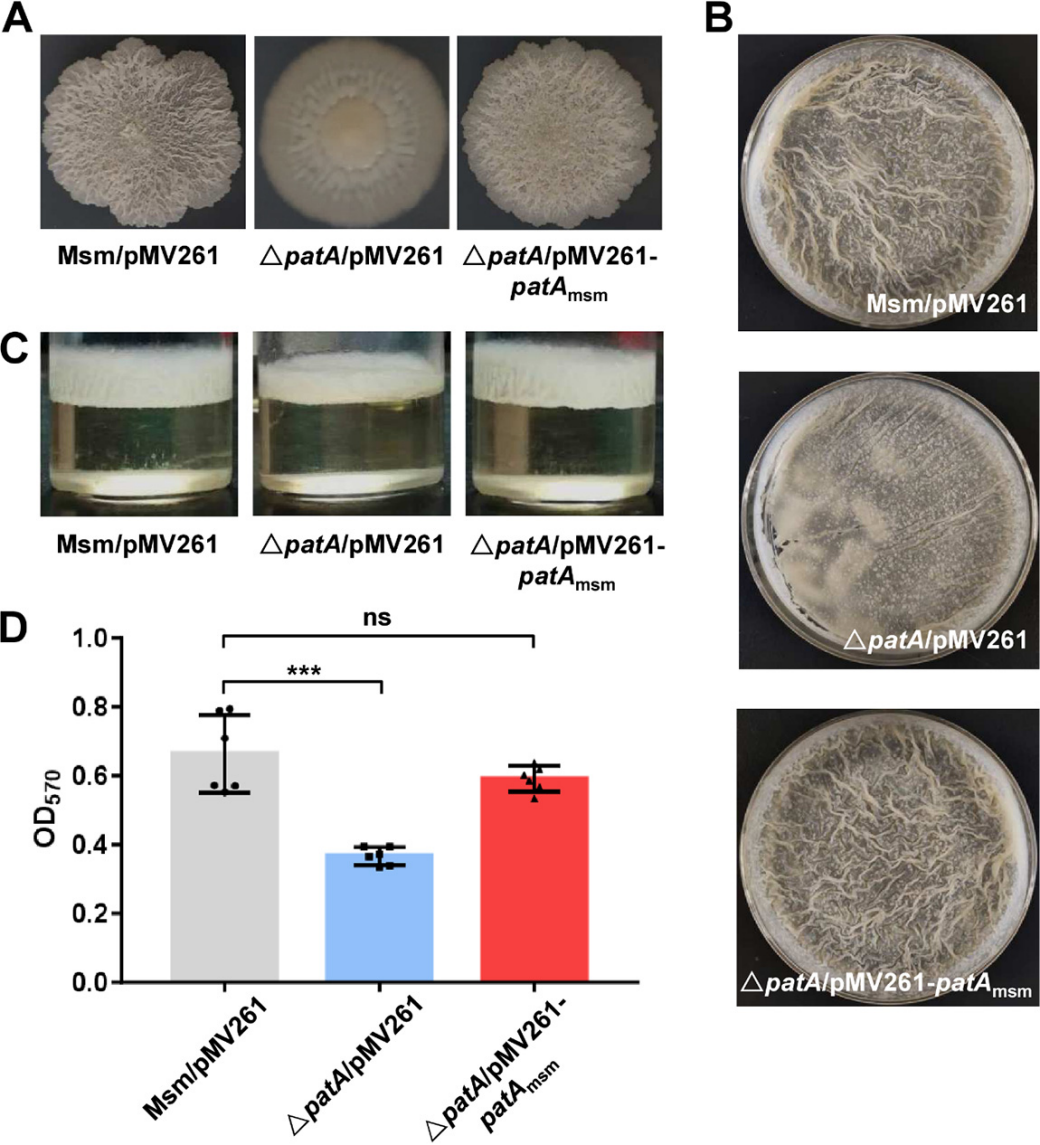
PatA positively regulates biofilm formation by M. smegmatis. (A) Effect of the *patA* deletion on mycobacterial spot colony morphology. From the left to the right are the M. smegmatis/pMV261 (Msm/pMV261), Δ*patA*/pMV261, and Δ*patA*/pMV261-*patA_msm_* strains grown on 7H10 medium plates. (B) Effect of the *patA* deletion on the surface morphology of the biofilm at the air-liquid interface. From the top to the bottom are the M. smegmatis/pMV261, Δ*patA*/pMV261, and Δ*patA*/pMV261-*patA_msm_* strains, respectively. (C) Biofilm thickness. From the left to the right are the M. smegmatis/pMV261, Δ*patA*/pMV261, and Δ*patA/*pMV261-*patA_msm_* strains, respectively. (D) Biofilm quantification by crystal violet staining. *** represents a significant difference between two groups at the level of a *P* value of <0.001 (by two-tailed Student’s *t* test). Data are expressed as the means ± standard deviations (SD) from six biological replicates. ns, not significant.

This study mainly investigated the changes in the colony surface morphology of a transposon insertion mutant of Mycolicibacterium smegmatis. The mutant strain displayed a smoother surface than the wild-type strain on 7H10 medium plates. The transposon insertion-induced mutation gene was identified as *patA* by sequencing of the insertion site. Next, we constructed a *patA* deletion mutant strain in M. smegmatis to examine the changes in the bacterial phenotypes. A significant difference in the spot colony morphologies of cells on 7H10 medium plates was observed between the wild-type and *patA*-deleted strains (Fig. 1A). The wild-type strain presented a typical wrinkled surface, whereas the *patA*-deleted strain had a relatively smooth surface and lacked a wrinkled surface. When the *patA* gene was expressed in the *patA*-deleted M. smegmatis strain, the complemented strain restored a spot colony morphology similar to that of the wild-type strain (Fig. 1A). These results suggested that PatA was involved in regulating the spot colony morphology of M. smegmatis.

Furthermore, we examined the effects of PatA on biofilm formation by M. smegmatis. As shown in Fig. 1B, the wild-type and *patA*-complemented strains formed significantly wrinkled pellicles at the air-liquid interface in M63 medium. In contrast, the *patA*-deleted strain formed a fragile film without the typical reticulation of the wild-type biofilm. Furthermore, the biofilm of the *patA*-deleted strain was thinner than those of the wild-type and *patA*-complemented strains (Fig. 1C), and this phenotype was further confirmed by biofilm quantification by crystal violet staining (Fig. 1D). These results indicated that PatA played an essential role in biofilm formation by mycobacteria.

### PatA positively regulates stress resistance in M. smegmatis.

Bacterial biofilms can improve their tolerance to environmental stresses such as antibiotics and oxidative stress (16). Considering that biofilm formation is usually associated with bacterial stress resistance, we examined the effects of the *patA* deletion on the stress resistance of M. smegmatis. As shown in Fig. S1A in the supplemental material, no growth difference was observed among the three strains without environmental stress. However, the bacterial counts of the *patA*-deleted strain were significantly lower than those of the wild-type and *patA*-complemented strains at 24 h and 28 h under environmental stress, including rifampicin (RIF), streptomycin (STR), and hydrogen peroxide (H_2_O_2_) treatments, which indicated that *patA* could play an important role in mycobacterial stress resistance (Fig. S1B to S1D).

Our results implied that the biofilm reduction caused by the *patA* deletion weakened the stress resistance of M. smegmatis, based on which it could be concluded that PatA positively regulated the stress resistance of M. smegmatis.

### PatA negatively regulates INH resistance in M. smegmatis.

**FIG 2:**
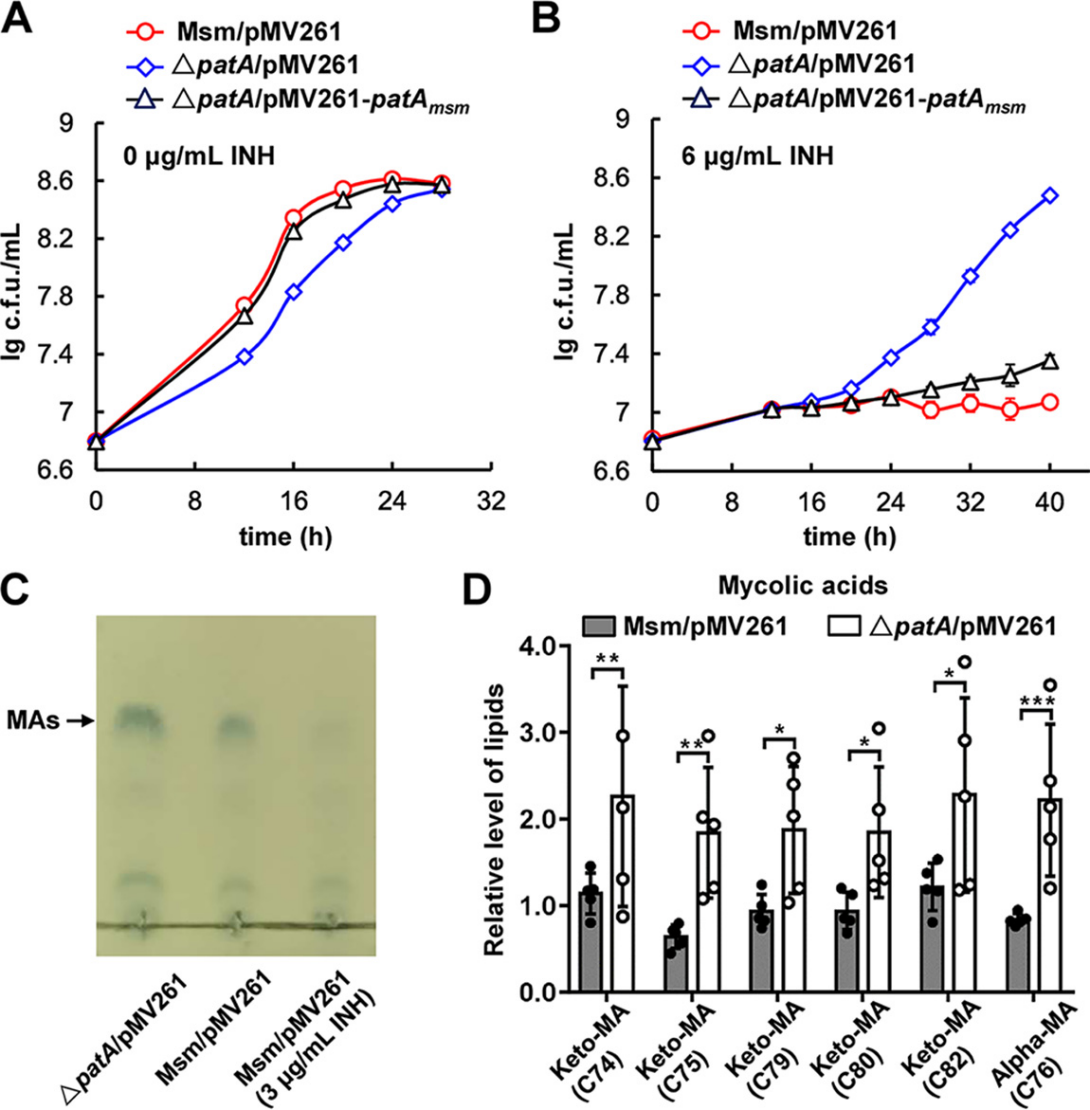
PatA negatively regulates mycolic acid synthesis and INH resistance in M. smegmatis. (A and B) Representative growth curves of the M. smegmatis/pMV261, Δ*patA*/pMV261, and Δ*patA*/pMV261-*patA_msm_* strains grown in 7H9 medium without INH (A) and in 7H9 medium supplemented with 6 μg/mL INH (B). Data are expressed as the means ± SD from three biological replicates. (C) Images of mycolic acids (MAs) in M. smegmatis upon INH stress. Mycolic acids were extracted. The samples were separated by TLC and imaged by spraying phosphomolybdic acid. From the left to the right are the Δ*patA*/pMV261 strain, the M. smegmatis/pMV261 strain, and the M. smegmatis/pMV261 strain under stress with 3 μg/mL INH. (D) Contents of various mycolic acids in the M. smegmatis/pMV261 and Δ*patA*/pMV261 strains.

As mentioned above, there was a positive correlation between PatA and mycobacterial stress resistance. However, under INH stress, the *patA*-deleted strain exhibited unexpected responses. Our bacterial growth curve showed that the bacterial counts of the *patA*-deleted mutant strain were slightly lower than those of the wild-type and *patA*-complemented strains without exogenous stress (Fig. 2A). Interestingly, under stress induced by 6 μg/mL INH, the wild-type strain was completely inhibited, but the *patA*-deleted strain seemed to be insensitive to INH stress (Fig. 2B). When the *patA* gene was expressed in the *patA*-deleted strain, the complemented strain exhibited growth inhibition that was similar to that of the wild-type strain (Fig. 2B), suggesting that PatA was important for mycobacterial resistance to INH stress.

Although biofilm formation by the *patA-*deleted strain was reduced, its INH resistance was significantly enhanced compared with that of the wild-type strain. INH has been reported to inhibit mycolic acid biosynthesis (22). Therefore, we speculated that PatA could affect the synthesis of mycolic acids. To verify this speculation, we extracted mycolic acids, analyzed them by thin-layer chromatography (TLC), and found that the mycolic acid content in the wild-type strain under INH stress was significantly lower than that without INH (Fig. 2C), indicating the inhibitory effect of INH on mycolic acid synthesis. Notably, the mycolic acid content in the *patA*-deleted strain was significantly higher than that in the wild-type strain (Fig. 2C), which was further supported by lipidomics data (Fig. 2D). In order to further investigate the related regulatory pathway, we performed proteomics analysis (data not shown). Interestingly, the *patA* deletion led to no significant changes in the protein levels in the FAS pathway, reported to be the only mycolic acid synthesis pathway, indicating that PatA-mediated mycolic acid synthesis is an unknown pathway.

Therefore, the *patA* deletion promoted the synthesis of mycolic acids, which could effectively counteract INH’s inhibition of mycolic acid synthesis in mycobacteria. Overall, PatA negatively regulated mycolic acid synthesis, thus leading to the INH resistance of the *patA-*deleted strain. The PatA-mediated mycolic acid synthesis pathway represents a hitherto-unknown synthesis pathway in mycobacteria.

### PatA protein homologs are conserved in mycobacteria.

**FIG 3:**
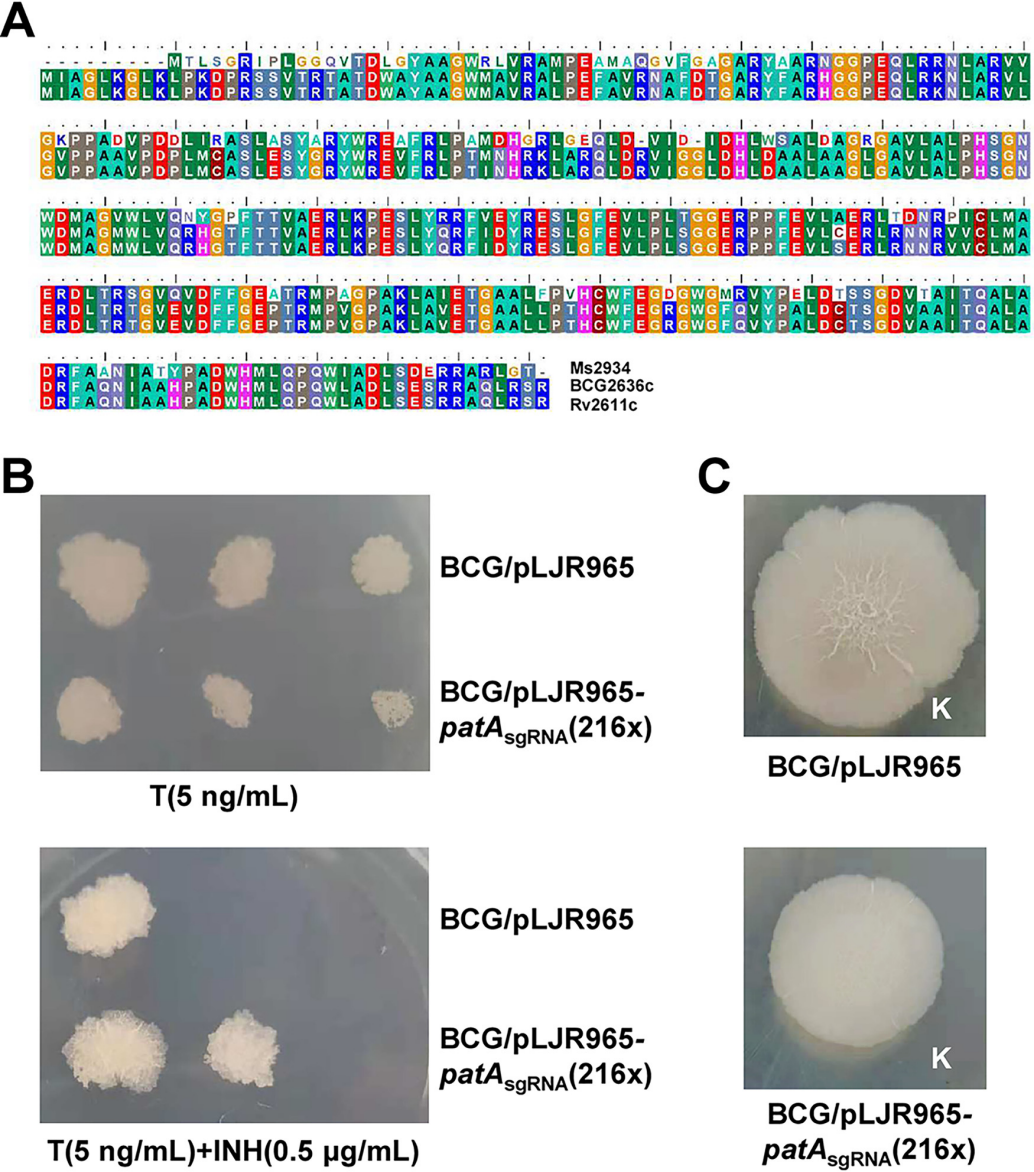
PatA_mbb_ negatively regulates INH resistance in M. bovis bacillus Calmette-Guérin (BCG). (A) PatA amino acid sequence alignment of M. smegmatis, the M. bovis BCG vaccine bacterium, and M. tuberculosis H37Rv. The conserved sequence is accentuated by filled backgrounds. Sequence alignment was performed using BioEdit software. (B) Colony morphologies of logarithmic-phase cultures of the BCG/pLJR965 and BCG/pLJR965-*patA*_sgRNA_(216 X) strains after serial dilutions on 7H10 medium plates supplemented with 5 ng/mL ATc [T(5 ng/mL)] or 5 ng/mL ATc plus 0.5 μg/mL INH. (C) Surface morphologies of the BCG/pLJR965 and BCG/pLJR965-*patA*_sgRNA_(216 X) strains in logarithmic phase on 7H10 medium plates without ATc.

Next, we investigated the conservation of PatA among several important mycobacterial species, including M. tuberculosis and the Mycobacterium bovis bacillus Calmette-Guérin (BCG) vaccine bacterium. The PatA protein is encoded by Rv2611c and BCG2636c in these two mycobacterial species, respectively. Rv2611c (M. tuberculosis PatA [PatA_mtu_]) and BCG2636c (M. bovis BCG PatA [PatA_mbb_]) were basically identical and showed 74.2% and 73.9% identities to M. smegmatis PatA (PatA_msm_), respectively (Fig. 3A). Furthermore, we attempted to construct a *patA*_mbb_-deleted strain through a series of homologous-recombination inductions, but we failed. Considering the essentiality of *patA*_mtu_ for M. tuberculosis (21), we speculated that *patA*_mbb_ was also essential for its growth. So we used CRISPR interference (CRISPRi) to silence the *patA*_mbb_ gene. As expected, under induction with anhydrous tetracycline (ATc), strains with low *patA*_mbb_ inhibition multiples grew slightly slower than the wild-type strains, while those with high *patA*_mbb_ inhibition multiples failed to survive (Fig. S2), suggesting that *patA*_mbb_ was required for BCG survival. Next, the relationship between PatA_mbb_ and INH resistance was investigated by spot colony morphology observations with and without INH. As shown in Fig. 3B, under induction with 5 ng/mL ATc, the colonies of the BCG *patA*-CRISPRi strain were smaller than those of the wild type, while the opposite results were observed under induction with 5 ng/mL ATc plus 0.5 μg/mL INH, indicating that PatA_mbb_ negatively regulated INH resistance in BCG. Furthermore, a significant difference was observed between the spot colony morphologies of the BCG wild-type strain and the *patA*-CRISPRi strains on 7H10 medium plates. Compared with the BCG wild-type strains, the *patA*-CRISPRi strains exhibited a smooth surface and grew slowly (Fig. 3C), which was similar to what we observed for M. smegmatis.

Taken together, these results indicated that PatA was not only homologous in amino acid sequence to BCG and M. smegmatis but also functionally conserved.

### PatA_mtu_ negatively regulates INH resistance in M. smegmatis.

**FIG 4:**
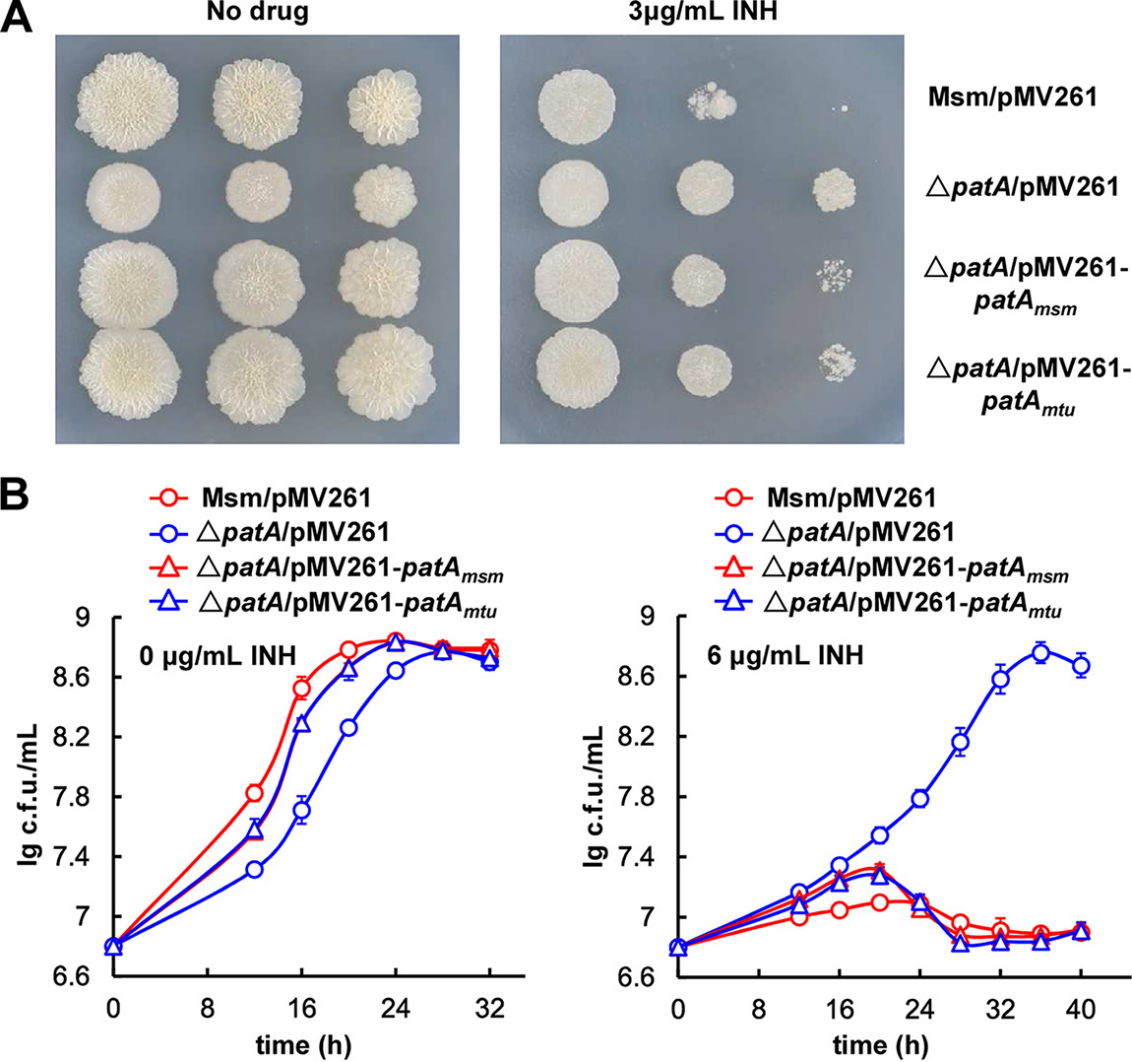
PatA_mtu_ negatively regulates INH resistance in M. smegmatis. (A) Growth status of the M. smegmatis/pMV261, Δ*patA*/pMV261, Δ*patA*/pMV261-*patA_msm_*, and Δ*patA*/pMV261-*patA_mtu_* strains after serial dilutions on 7H10 medium plates with (right) or without (left) 3 μg/mL INH. (B) Representative growth curves of the M. smegmatis/pMV261, Δ*patA*/pMV261, Δ*patA*/pMV261-*patA_msm_*, and Δ*patA/*pMV261-*patA_mtu_* strains grown in 7H9 medium with or without 6 μg/mL INH. Data are expressed as the means ± SD from three biological replicates.

Considering the homology between PatA_mtu_ and PatA_msm_, we expressed *patA*_mtu_ in the *patA*_msm_-deleted strain and defined it as the cross-complemented strain to investigate the roles of PatA_mtu_ in mycobacterial INH resistance. As shown in Fig. 4A, the growth status of the cross-complemented strain was the same as those of the wild-type strain and the homologous complemented strain, and the colonies of these three strains were larger than that of the *patA*-deleted strain on 7H10 plates without INH. However, on plates with 3 μg/mL INH, the growth status of these strains was completely the opposite, and the sensitivity of the cross-complemented strain to INH was restored to the same level as that of the complemented strain, while the *patA*-deleted strain withstood the INH stress and grew well. The subsequently constructed growth curve further confirmed these results (Fig. 4B). The above-described results indicated that PatA_mtu_ negatively regulated INH resistance in M. smegmatis, which was in line with the results for PatA_msm_.

In summary, like the complemented strain, the cross-complemented strain could regain sensitivity to INH, which indicated that PatA was functionally conserved and that it could regulate INH resistance in several mycobacterial species, including M. tuberculosis and BCG.

### PatA_mtu_ and PatA_msm_ have the same functions in regulating growth, colony surface morphology, and biofilm formation in mycobacteria.

**FIG 5:**
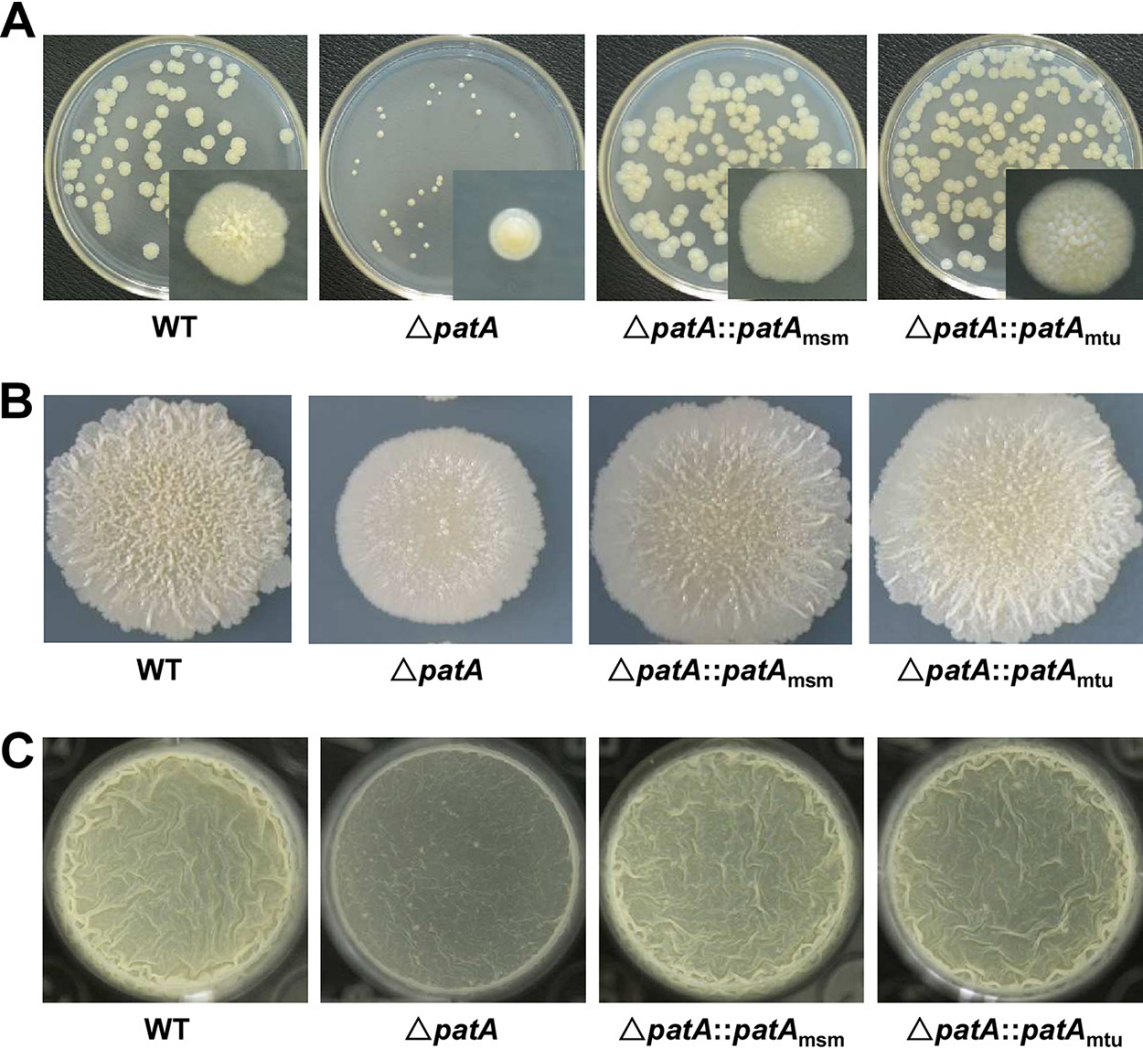
PatA_mtu_ positively regulates the growth, colony surface morphology, and biofilm formation of M. smegmatis. (A) M. smegmatis single-colony surface morphology. Logarithmic-phase cultures were diluted 1 million times for the M. smegmatis/pMV261, Δ*patA*/pMV261, Δ*patA*/pMV261-*patA_msm_*, and Δ*patA*/pMV261-*patA_mtu_* strains on 7H10 medium plates. (B) Spot colony morphologies of the M. smegmatis/pMV261, Δ*patA*/pMV261, Δ*patA*/pMV261-*patA_msm_*, and Δ*patA*/pMV261-*patA_mtu_* strains in logarithmic phase on 7H10 medium plates. (C) Surface morphologies of biofilms at the air-liquid interface. From the top to the bottom are the M. smegmatis/pMV261, Δ*patA*/pMV261, Δ*patA*/pMV261-*patA_msm_*, and Δ*patA*/pMV261-*patA_mtu_* strains. WT, wild type.

We further examined the regulatory effect of PatA_mtu_ on the growth, colony surface morphology, and biofilm formation of M. smegmatis. As shown in Fig. 5A, the cross-complemented strain compensated for the smaller colony size caused by the *patA* deletion and reobtained a wrinkled surface morphology similar to that of the wild-type strain. Likewise, we further confirmed these results by spot colony morphology observations (Fig. 5B), experiments with streaking (Fig. S3) onto 7H10 medium plates, and air-liquid interface biofilm observations in M63 medium (Fig. 5C).

In conclusion, the cross-complemented strain could compensate for the phenotypic changes in the *patA*-deleted strain. These results indicated that PatA_mtu_ and PatA_msm_ shared functions in regulating mycobacterial growth, colony surface morphology, and biofilm formation.

### PatA mediates biofilm formation by positively regulating the synthesis of lipids (except mycolic acids).

**FIG 6:**
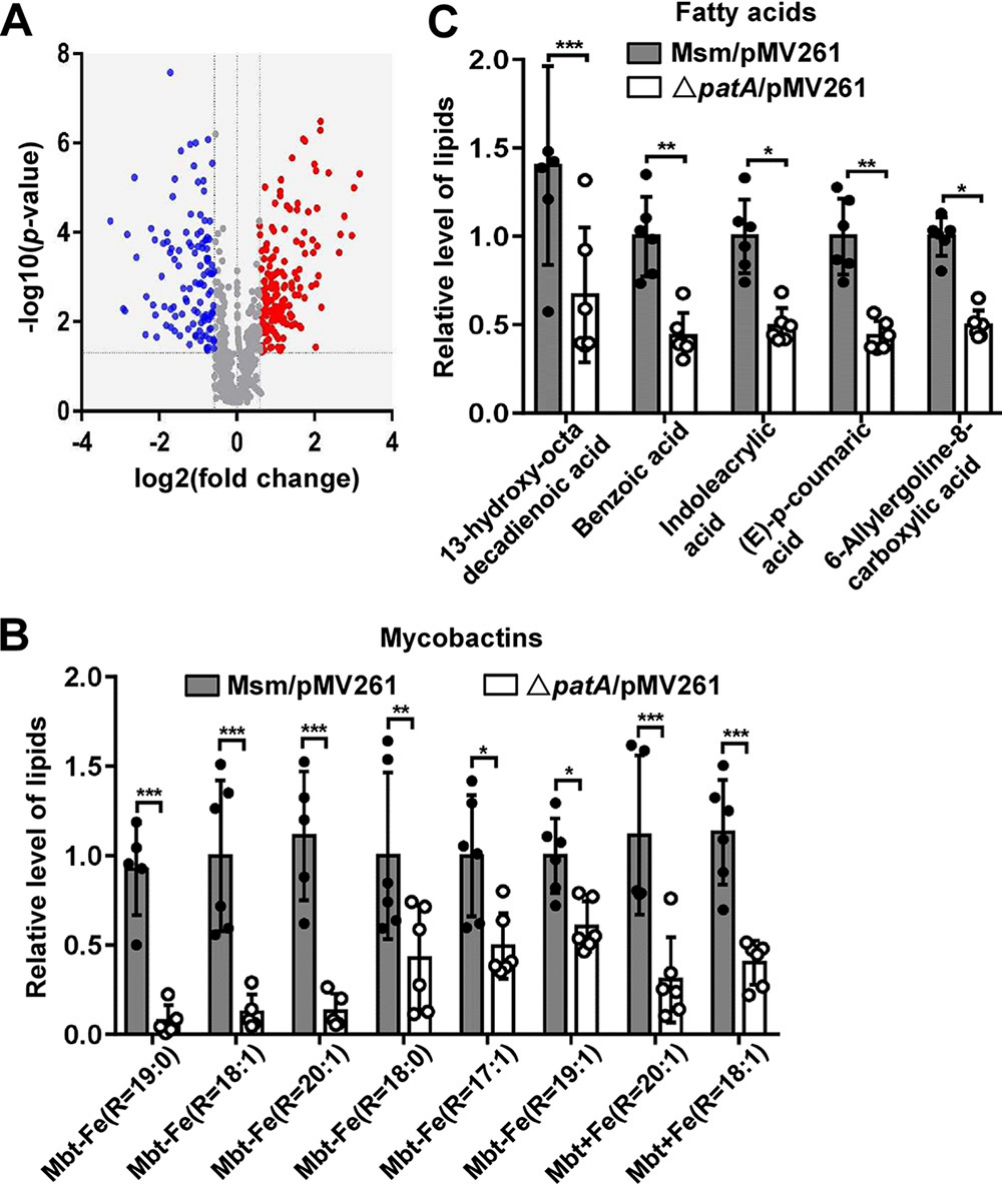
Lipidomics analysis of the *patA*-deleted mutant strain of M. smegmatis. (A) Volcano diagram of lipid composition differences between the M. smegmatis/pMV261 and Δ*patA*/pMV261 strains determined by lipidomics assays. The red spots indicate significantly upregulated genes. The blue and gray spots denote downregulated genes and those without significant changes, respectively. (B) Mycobactin (Mbt) contents in the M. smegmatis/pMV261 and Δ*patA*/pMV261 strains. (C) Fatty acid contents in the M. smegmatis/pMV261 and Δ*patA*/pMV261 strains. *, *P < *0.05; **, *P < *0.01; ***, *P < *0.001 (by two-tailed Student’s *t* test). Data are expressed as the means ± SD from six biological replicates.

The synthesis of lipids can affect the mycobacterial biofilm formation (23–25). Considering the effects of PatA on mycobacterial biofilm formation, we hypothesized that PatA might regulate the synthesis of lipids in mycobacteria. To verify this hypothesis, we further analyzed the lipidomics data. As shown in Fig. 6A, the contents of 112 lipids were significantly increased and those of 156 lipids were significantly decreased in the *patA*-deleted strain compared with those in the wild-type strain. Further analysis showed that the contents of different types of mycobactins were also significantly decreased in the *patA*-deleted strain (Fig. 6B), and the contents of five different types of fatty acids showed the same changing trend as that for the above-mentioned mycobactins (Fig. 6C).

Taken together, the *patA* deletion led to changes in the contents of various types of lipids in M. smegmatis, among which the reductions in the contents of mycobactins and fatty acids were mainly responsible for the decline in biofilm formation. These results indicated that PatA mediated biofilm formation by positively regulating the synthesis of lipids (except mycolic acids).

## DISCUSSION

The crystal structure and catalytic function of PatA have been extensively studied in bacteria (26–28), but whether PatA regulates mycolic acid synthesis and biofilm formation and affects stress resistance in mycobacteria is unclear, and the involved mechanisms remain to be explored. In the present study, we identified multiple functions of PatA and found that the *patA* deletion promoted the synthesis of mycolic acids through unclear mechanisms, which effectively counteracted INH’s inhibition of mycolic acid synthesis in mycobacteria, eventually leading to the INH resistance of the *patA*-deleted mutant. In addition, PatA regulated the synthesis of lipids other than mycolic acids to maintain biofilm formation and stress resistance in M. smegmatis, and PatA was highly conserved and showed the same physiological functions in several mycobacterial species. This study expanded the pathway of mycolic acid synthesis and revealed the correlation between lipid metabolism and bacterial physiological phenotypes.

Mycolic acids are some of the most abundant components of the cell wall of mycobacteria, and mycolic acids are synthesized mainly via the well-characterized FAS-I and FAS-II biosynthetic pathways (8, 9, 29, 30). In the present study, the *patA* deletion was found to promote the synthesis of mycolic acids to counteract INH’s inhibition of the synthesis of mycolic acids. The FAS pathway has been reported to be the only pathway for mycolic acid synthesis. However, this study discovered for the first time the PatA-mediated mycolic acid synthesis pathway, which might represent a novel synthesis pathway in mycobacteria. It is well known that PatA is an essential acyltransferase for the biosynthesis of PIMs (21), and PIMs are important precursors for the synthesis of lipomannan (LM) and lipoarabinomannan (LAM). Thus, the substances in the LM and LAM synthetic pathways might contribute to the synthesis of mycolic acids, which remains to be further investigated. Our data revealed that PatA negatively regulated mycolic acids, thus inducing mycobacterial INH resistance.

INH is one of the first line anti-tuberculosis drugs (11), and its bactericidal mechanism is mainly through acting on its target enoyl acyl carrier protein reductase (InhA), and InhA is an enzyme involved mainly in fatty acid synthesis and mycolic acid biosynthesis (12, 31). INH resistance arises primarily from mutations of the mycobacterial catalase-peroxidase (KatG) or InhA (32). In the present study, the mutation of PatA also resulted in INH resistance in mycobacteria, implying that PatA might be a new target of INH, which needs to be verified by further work. In addition, PatA is essential in M. tuberculosis, implying that PatA is a potential target for drug development against this pathogenic microorganism.

The changes in certain types of lipid content caused by the *patA* deletion are the main factors that directly affect mycobacterial biofilm formation. Our lipidomics results showed that the *patA* deletion led to significant changes in hundreds of substances, thus resulting in some changes in the physiological phenotypes of mycobacteria. M. tuberculosis has to resist host defenses during infections, and lipids of this pathogen are important for mediating immune suppression (33). Our results implied that PatA was associated with the virulence and immunity of pathogenic bacteria, but more evidence is needed.

**FIG 7:**
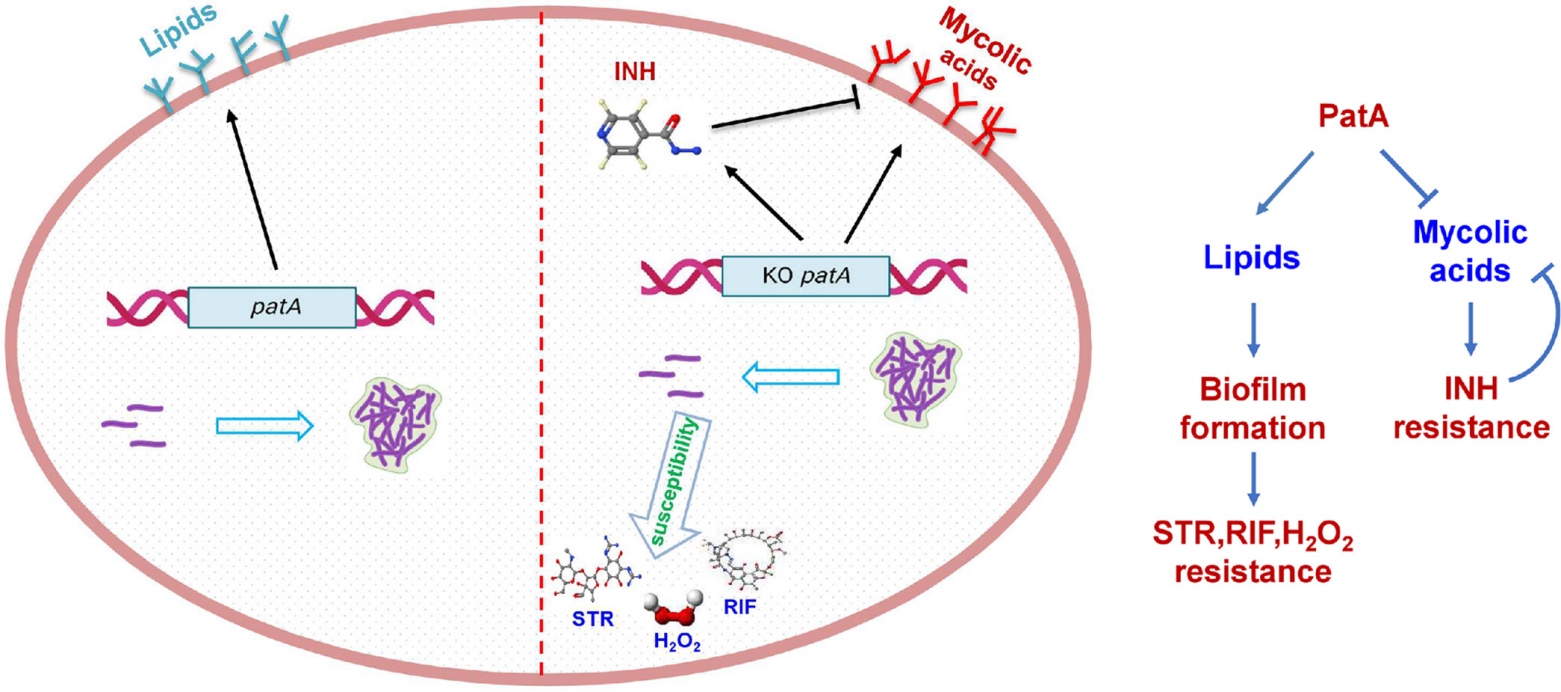
Schematic diagram of the molecular mechanism by which PatA mediates isoniazid (INH) resistance by regulating a novel synthetic pathway for mycolic acids and regulates biofilm formation in mycobacteria. (Left) Pattern diagram of this study. (Right) Regulatory pathway for PatA. Arrows indicate positive regulatory effects, and T-shaped arrows indicate negative regulatory effects. STR, streptomycin; H_2_O_2_, hydrogen peroxide; RIF, rifampicin; KO, knockout.

In summary, our results indicated that PatA affected INH resistance and biofilm formation by regulating the lipid contents in the mycobacterial cell wall. As shown in Fig. 7, after the *patA* deletion, the contents of many types of lipids (except mycolic acids) in the mycobacterial cell wall decreased, thus leading to reductions in biofilm formation and stress resistance. However, the increased concentrations of mycolic acids induced by the *patA* deletion effectively counteracted INH’s inhibition of mycolic acid synthesis, thereby resulting in the increase in the resistance of the mutant to INH. This study reveals a PatA-mediated mycolic acid synthetic pathway, and our findings provide a new perspective for research on the drug resistance mechanisms of M. tuberculosis and offer a valuable reference for drug development against TB.

## MATERIALS AND METHODS

### Bacterial strains and media.

M. smegmatis mc^2^155 and the M. bovis bacillus Calmette-Guérin (BCG) vaccine bacterium were grown at 37°C in 7H9 medium supplemented with 0.05% (vol/vol) Tween 80 and 0.2% (vol/vol) glycerol. M63 medium was used for experiments with the air-liquid interface, and the ingredients of this culture medium include 6.805 g of KH_2_PO_4_, 0.992 g of (NH_4_)_2_SO_4_, 0.124 g of MgSO_4_, 2.104 g of KOH, and 39 μL of a 100 mM FeSO_4_ solution per 500 mL of water for cloning procedures. Escherichia coli strain DH5α was grown in Luria-Bertani (LB) medium. When required, hygromycin was used at a final concentration of 50 μg/mL for M. smegmatis. Kanamycin was used at a final concentration of 30 μg/mL for M. smegmatis and E. coli.

### Construction of the M. smegmatis *patA-*deleted mutant and complemented strains.

A pMind suicide plasmid (34) carrying a hygromycin resistance gene was constructed, and a *lacZ* gene was inserted to confer sensitivity to X-gal (5-bromo-4-chloro-3-indolyl-β-d-galactopyranoside). The knockout of the *patA* gene in M. smegmatis mc^2^155 was performed as described previously (35). The constructed recombinant plasmid (pMV261-*patA*) was electroporated into the M. smegmatis mc^2^155 *patA*-deleted mutant in the competent state, and finally, the complemented strain was obtained. Cross-complementary strains were obtained in the same way.

### Culture of biofilm at the air-liquid interface.

Different recombinant M. smegmatis strains were cultured in 7H9 medium to mid-log phase and resuspended in M63 medium. The suspended solids were inoculated into fresh M63 medium, and the initial concentration was controlled to OD_600_ = 0.1. Subsequently, the cells were incubated at 22°C for 4 days.

### Quantification of biofilm.

Different recombinant M. smegmatis strains were cultured in 7H9 medium to mid-log phase and then resuspended in M63 medium. The suspended solids were inoculated into fresh M63 medium, and the initial concentration was controlled to OD_600_ = 0.1. Subsequently, the cells were cultured at 80 rpm/min at 37°C for 1.5 days. The bacterial medium was drained, and the remaining biofilm was rinsed twice with double-distilled water (ddH_2_O). Next, 120 μL of 1% crystal violet was added for staining for 30 min. After that, the biofilm was washed three times with 200 μL ddH_2_O. Two hundred microliters of an ethanol-acetone mixture was added, and the mixture was dissolved at room temperature for 5 min. Finally, the absorbance was measured at 570 nm. All results were replicated in 6 biological replicates, and error values were calculated.

### Determination of mycobacterial growth.

The target recombinant strains were grown to mid-log phase in 7H9 medium containing 30 μg/mL kanamycin. After harvest, the strains were resuspended in fresh medium. Next, they were added to 100 mL of fresh 7H9 medium containing 30 μg/mL of kanamycin and stress inducers at the indicated concentrations, and the initial concentration was controlled to OD_600_ = 0.1. After that, the cultures were grown at 37°C in a shaker at 160 rpm. The samples were taken at different time points, diluted, and spread onto 7H10 medium plates to count the CFU. Three parallel experiments were performed for each sample.

### Extraction and analysis of mycolic acids.

Strains were grown in 7H9 medium at 37°C with shaking at 160 rpm. All strains were harvested and washed in a phosphate-buffered saline (PBS) solution when wild-type strains were grown to mid-log phase (OD_600_ = 1.0). Next, 50 mg of bacterial cells was resuspended in 1 mL of a chloroform-methanol solution (2:1), followed by 3 repeats of sonication (1-min duration) and vibration. Next, the cell debris was collected after transfer to a 15-mL centrifuge tube and air dried. After that, 5 mL of a 5% (wt/vol) aqueous tetrabutylammonium hydroxide (TBAH) solution (Aladdin) was added to the tube, and the tube was heated at 100°C for 4 h. After cooling, the mixture was transferred to another tube containing 5 mL dichloromethane and 62.5 μL iodomethane, and the mixture was shaken for 1 h. For each sample, the bottom phase containing lipids was collected and air dried overnight in a fume hood. Lipids were analyzed by TLC, developed in hexane-ethyl acetate (95:5), and imaged by spraying 10% (wt/vol) phosphomolybdic acid in ethanol and heating it.

### Lipidomics analysis.

The lipid component was extracted from the biofilm of the strain after culture. Next, Sciex ExionLC was used as the supernatant for chromatographic analysis. After ultraperformance liquid chromatography (UPLC) separation, a triple-time-of-flight (TOF) 5600 instrument with an electrospray ionization (ESI) source was used for mass spectrometry analysis. For the positive ion acquisition mode, the information dependent acquisition (IDA) highly sensitive scanning mode was adopted, with a quality scanning range of 50 to 1,200 Da and a scanning time of 20 min. For the negative ion acquisition mode, the IDA highly sensitive scanning mode was adopted, with a quality scanning range of 50 to 1,200 Da and a scanning time of 20 min. The original deactivation data file was imported into Progenesis QI software, and peak alignment was carried out according to the retention time, mass/charge ratio, mass deviation, and other parameters for each compound. The processed mass spectrum data were matched with NIST (https://www.nist.gov/), Lipidmaps (https://www.lipidmaps.org), and Mtb LipidDB (36) data, comparing the databases of lipids for qualitative and quantitative data results. Lipidomics was performed by Beijing Novogene Biotechnology Corporation.

### Proteomic analysis.

Strains were grown in 7H9 medium at 37°C with shaking at 160 rpm. All strains were harvested and washed with a PBS solution when wild-type strains were grown to mid-log phase. After taking the appropriate amount of samples for grinding, the protein was extracted, and its concentration was determined. The protein samples were enzymolyzed in equal amounts overnight, dithiothreitol (DTT) was then added, and the samples were reduced at 56°C for 30 min. After that, iodoacetamide (IAA) was added at 11 mM, and the mixture was incubated at room temperature for 15 min away from light. The peptides were dissolved in a liquid chromatography mobile phase and separated using the Easy-nLC 1200 ultrahigh-performance liquid-phase system. After that, the peptides were separated by the ultrahigh-performance liquid-phase system, ionized by injection into an nanometer spray ionization (NSI) ion source, and then analyzed by Orbitrap Exploris 480 mass spectrometry. The ion source voltage was set to 2.3 kV, and the high-field asymmetric waveform ion mobility spectrometry (FAIMS) compensation voltages (CVs) were set to −45 V and −65 V. The parent ions and secondary fragments of the peptide were detected and analyzed using a high-resolution Orbitrap instrument. The scanning range for primary mass spectrometry was set from 400 to 1,200 *m/z*, and the scanning resolution was set to 60,000. The scanning range for two-stage mass spectrometry was fixed at 110 *m/z*, the two-stage scanning resolution was set to 15,000, and the turbo tandem mass tag (TMT) was set to off. Proteomics was performed by Hangzhou Jingjie Biotechnology Corporation.

### CRISPRi assay.

Cloning of the CRISPRi plasmid was performed as described previously using Addgene plasmid 115163 (37). In short, the CRISPRi plasmid was digested by the endonuclease BsmBI and gel purified. Each pair of single guide RNAs (sgRNAs), two complemented oligonucleotides, was annealed and ligated into the digested plasmid. The successfully constructed recombinant plasmids were electroporated into the BCG wild-type strain to obtain CRISPRi strains. At the same time, the empty plasmid was transformed as a control. The inhibition ratio of related genes in the CRISPRi strain can be adjusted by using different concentrations of ATc.

### Data availability.

The mass spectrometry proteomics data have been deposited to the ProteomeXchange Consortium via the PRIDE (38) partner repository with the data set identifier PXD038853.

## References

[1] Houben RMGJ, Dodd PJ. 2016. The global burden of latent tuberculosis infection: a re-estimation using mathematical modelling. PLoS Med 13:e1002152. doi:10.1371/journal.pmed.1002152. 27780211 PMC5079585

[2] Savkova K, Huszar S, Barath P, Pakanova Z, Kozmon S, Vancova M, Tesarova M, Blasko J, Kalinak M, Singh V, Kordulakova J, Mikusova K. 2021. An ABC transporter Wzm-Wzt catalyzes translocation of lipid-linked galactan across the plasma membrane in mycobacteria. Proc Natl Acad Sci USA 118:e2023663118. doi:10.1073/pnas.2023663118. 33879617 PMC8092408

[3] World Health Organization. 2022. WHO global tuberculosis report. World Health Organization, Geneva, Switzerland. https://www.who.int/teams/global-tuberculosis-programme/tb-reports/global-tuberculosis-report-2022.

[4] Garcia-Vilanova A, Chan J, Torrelles JB. 2019. Underestimated manipulative roles of *Mycobacterium tuberculosis* cell envelope glycolipids during infection. Front Immunol 10:2909. doi:10.3389/fimmu.2019.02909. 31921168 PMC6930167

[5] Gago G, Diacovich L, Gramajo H. 2018. Lipid metabolism and its implication in mycobacteria-host interaction. Curr Opin Microbiol 41:36–42. doi:10.1016/j.mib.2017.11.020. 29190491 PMC5862736

[6] Verschoor JA, Baird MS, Grooten J. 2012. Towards understanding the functional diversity of cell wall mycolic acids of *Mycobacterium tuberculosis*. Prog Lipid Res 51:325–339. doi:10.1016/j.plipres.2012.05.002. 22659327

[7] Bhat ZS, Rather MA, Maqbool M, Lah HU, Yousuf SK, Ahmad Z. 2017. Cell wall: a versatile fountain of drug targets in *Mycobacterium tuberculosis*. Biomed Pharmacother 95:1520–1534. doi:10.1016/j.biopha.2017.09.036. 28946393

[8] Marrakchi H, Laneelle MA, Daffe M. 2014. Mycolic acids: structures, biosynthesis, and beyond. Chem Biol 21:67–85. doi:10.1016/j.chembiol.2013.11.011. 24374164

[9] Quemard A. 2016. New insights into the mycolate-containing compound biosynthesis and transport in mycobacteria. Trends Microbiol 24:725–738. doi:10.1016/j.tim.2016.04.009. 27268593

[10] Meyer H, Mally J. 1912. Über Hydrazinderivate der Pyridincarbonsäuren. Monatsh Chem Verwandte Teile Anderer Wiss 33:393–414. doi:10.1007/BF01517946.

[11] Whitney JB, Wainberg MA. 2002. Isoniazid, the frontline of resistance in *Mycobacterium tuberculosis*. McGill J Med 6:114–123. doi:10.26443/mjm.v6i2.686.

[12] Stanley SA, Kawate T, Iwase N, Shimizu M, Clatworthy AE, Kazyanskaya E, Sacchettini JC, Ioerger TR, Siddiqi NA, Minami S, Aquadro JA, Grant SS, Rubin EJ, Hung DT. 2013. Diarylcoumarins inhibit mycolic acid biosynthesis and kill *Mycobacterium tuberculosis* by targeting FadD32. Proc Natl Acad Sci USA 110:11565–11570. doi:10.1073/pnas.1302114110. 23798446 PMC3710825

[13] Rella A, Farnoud AM, Del Poeta M. 2016. Plasma membrane lipids and their role in fungal virulence. Prog Lipid Res 61:63–72. doi:10.1016/j.plipres.2015.11.003. 26703191 PMC4733445

[14] O’Toole G, Kaplan HB, Kolter R. 2000. Biofilm formation as microbial development. Annu Rev Microbiol 54:49–79. doi:10.1146/annurev.micro.54.1.49. 11018124

[15] Flemming HC, Wingender J, Szewzyk U, Steinberg P, Rice SA, Kjelleberg S. 2016. Biofilms: an emergent form of bacterial life. Nat Rev Microbiol 14:563–575. doi:10.1038/nrmicro.2016.94. 27510863

[16] Venkatesan N, Perumal G, Doble M. 2015. Bacterial resistance in biofilm-associated bacteria. Future Microbiol 10:1743–1750. doi:10.2217/fmb.15.69. 26517598

[17] Kumar A, Alam A, Rani M, Ehtesham NZ, Hasnain SE. 2017. Biofilms: survival and defense strategy for pathogens. Int J Med Microbiol 307:481–489. doi:10.1016/j.ijmm.2017.09.016. 28950999

[18] Koo H, Allan RN, Howlin RP, Stoodley P, Hall-Stoodley L. 2017. Targeting microbial biofilms: current and prospective therapeutic strategies. Nat Rev Microbiol 15:740–755. doi:10.1038/nrmicro.2017.99. 28944770 PMC5685531

[19] Lianou A, Nychas GE, Koutsoumanis KP. 2020. Strain variability in biofilm formation: a food safety and quality perspective. Food Res Int 137:109424. doi:10.1016/j.foodres.2020.109424. 33233106

[20] Tenke P, Riedl CR, Jones GL, Williams GJ, Stickler D, Nagy E. 2004. Bacterial biofilm formation on urologic devices and heparin coating as preventive strategy. Int J Antimicrob Agents 23(Suppl 1):S67–S74. doi:10.1016/j.ijantimicag.2003.12.007. 15037330

[21] Boldrin F, Anso I, Alebouyeh S, Sevilla IA, Geijo M, Garrido JM, Marina A, Cioetto Mazzabo L, Segafreddo G, Guerin ME, Manganelli R, Prados-Rosales R. 2021. The phosphatidyl-*myo*-inositol dimannoside acyltransferase PatA is essential for *Mycobacterium tuberculosis* growth *in vitro* and *in vivo*. J Bacteriol 203:e00439-20. doi:10.1128/JB.00439-20. PMC8088522 33468587

[22] Vilcheze C, Wang F, Arai M, Hazbon MH, Colangeli R, Kremer L, Weisbrod TR, Alland D, Sacchettini JC, Jacobs WR, Jr. 2006. Transfer of a point mutation in *Mycobacterium tuberculosis inhA* resolves the target of isoniazid. Nat Med 12:1027–1029. doi:10.1038/nm1466. 16906155

[23] Trivedi A, Mavi PS, Bhatt D, Kumar A. 2016. Thiol reductive stress induces cellulose-anchored biofilm formation in *Mycobacterium tuberculosis*. Nat Commun 7:11392. doi:10.1038/ncomms11392. 27109928 PMC4848537

[24] Pacheco SA, Hsu F-F, Powers KM, Purdy GE. 2013. MmpL11 protein transports mycolic acid-containing lipids to the mycobacterial cell wall and contributes to biofilm formation in *Mycobacterium smegmatis*. J Biol Chem 288:24213–24222. doi:10.1074/jbc.M113.473371. 23836904 PMC3745366

[25] Xu H, Su Z, Li W, Deng Y, He Z-G. 2021. MmbR, a master transcription regulator that controls fatty acid β-oxidation genes in *Mycolicibacterium smegmatis*. Environ Microbiol 23:1096–1114. doi:10.1111/1462-2920.15249. 32985741

[26] Albesa-Jove D, Svetlikova Z, Tersa M, Sancho-Vaello E, Carreras-Gonzalez A, Bonnet P, Arrasate P, Eguskiza A, Angala SK, Cifuente JO, Kordulakova J, Jackson M, Mikusova K, Guerin ME. 2016. Structural basis for selective recognition of acyl chains by the membrane-associated acyltransferase PatA. Nat Commun 7:10906. doi:10.1038/ncomms10906. 26965057 PMC4792965

[27] Tersa M, Raich L, Albesa-Jove D, Trastoy B, Prandi J, Gilleron M, Rovira C, Guerin ME. 2018. The molecular mechanism of substrate recognition and catalysis of the membrane acyltransferase PatA from mycobacteria. ACS Chem Biol 13:131–140. doi:10.1021/acschembio.7b00578. 29185694

[28] Anso I, Basso L, Wang L, Marina A, Paez-Perez ED, Jager C, Gavotto F, Tersa M, Perrone S, Contreras FX, Prandi J, Gilleron M, Linster CL, Corzana F, Lowary TL, Trastoy B, Guerin ME. 2021. Molecular ruler mechanism and interfacial catalysis of the integral membrane acyltransferase PatA. Sci Adv 7:eabj4565. doi:10.1126/sciadv.abj4565. 34652941 PMC8519569

[29] PaweŁczyk J, Kremer L. 2014. The molecular genetics of mycolic acid biosynthesis. Microbiol Spectr 2:MGM2-0003-2013. doi:10.1128/microbiolspec.MGM2-0003-2013. 26104214

[30] Sacco E, Covarrubias AS, O’Hare HM, Carroll P, Eynard N, Jones TA, Parish T, Daffe M, Backbro K, Quemard A. 2007. The missing piece of the type II fatty acid synthase system from *Mycobacterium tuberculosis*. Proc Natl Acad Sci USA 104:14628–14633. doi:10.1073/pnas.0704132104. 17804795 PMC1976197

[31] Banerjee A, Dubnau E, Quemard A, Balasubramanian V, Um KS, Wilson T, Collins D, de Lisle G, Jacobs WR, Jr. 1994. *inhA*, a gene encoding a target for isoniazid and ethionamide in *Mycobacterium tuberculosis*. Science 263:227–230. doi:10.1126/science.8284673. 8284673

[32] Jiang Z, Lu Y, Liu Z, Wu W, Xu X, Dinnyes A, Yu Z, Chen L, Sun Q. 2022. Drug resistance prediction and resistance genes identification in *Mycobacterium tuberculosis* based on a hierarchical attentive neural network utilizing genome-wide variants. Brief Bioinform 23:bbac041. doi:10.1093/bib/bbac041. 35325021

[33] Ehrt S, Schnappinger D. 2007. *Mycobacterium tuberculosis* virulence: lipids inside and out. Nat Med 13:284–285. doi:10.1038/nm0307-284. 17342139

[34] Blokpoel MCJ, Murphy HN, O’Toole R, Wiles S, Runn ESC, Stewart GR, Young DB, Robertson BD. 2005. Tetracycline-inducible gene regulation in mycobacteria. Nucleic Acids Res 33:e22. doi:10.1093/nar/gni023. 15687380 PMC548381

[35] Li W, He Z-G. 2012. LtmA, a novel cyclic di-GMP-responsive activator, broadly regulates the expression of lipid transport and metabolism genes in *Mycobacterium smegmatis*. Nucleic Acids Res 40:11292–11307. doi:10.1093/nar/gks923. 23047950 PMC3526308

[36] Sartain MJ, Dick DL, Rithner CD, Crick DC, Belisle JT. 2011. Lipidomic analyses of *Mycobacterium tuberculosis* based on accurate mass measurements and the novel “Mtb LipidDB.” J Lipid Res 52:861–872. doi:10.1194/jlr.M010363. 21285232 PMC3073466

[37] Wong AI, Rock JM. 2021. CRISPR interference (CRISPRi) for targeted gene silencing in mycobacteria. Methods Mol Biol 2314:343–364. doi:10.1007/978-1-0716-1460-0_16. 34235662

[38] Perez-Riverol Y, Bai J, Bandla C, Garcia-Seisdedos D, Hewapathirana S, Kamatchinathan S, Kundu DJ, Prakash A, Frericks-Zipper A, Eisenacher M, Walzer M, Wang S, Brazma A, Vizcaino JA. 2022. The PRIDE database resources in 2022: a hub for mass spectrometry-based proteomics evidences. Nucleic Acids Res 50:D543–D552. doi:10.1093/nar/gkab1038. 34723319 PMC8728295

