# Supplementary material for: PatA Regulates Isoniazid Resistance by Mediating Mycolic Acid Synthesis and Controls Biofilm Formation by Affecting Lipid Synthesis in Mycobacteria
Source: Microbiol Spectr. 2023 May 22;11(3):e00928-23. doi: 10.1128/spectrum.00928-23 (PMC10269662; doi:10.1128/spectrum.00928-23)
Supplement: Supplemental file 1 — Fig. S1 to S3. Download spectrum.00928-23-s0001.docx, DOCX file, 3.0 MB [file spectrum.00928-23-s0001.docx]

**PatA regulates isoniazid resistance through mediating mycolic acid synthesis as a novel pathway, and controls biofilm formation by affecting lipid synthesis in mycobacteria**

**Kun Wang, Yimin Deng, Xujie Cui, Mengli Chen, Yanzhe Ou, Danting Li****, Minhao Guo, Weihui Li***

State Key Laboratory for Conservation and Utilization of Subtropical Agro-bioresources, College of Life Science and Technology, Guangxi University, Nanning 530004, China

*To whom correspondence should be addressed: State Key Laboratory for Conservation and Utilization of Subtropical Agro-bioresources, College of Life Science and Technology, Guangxi University, Nanning 530004, China

Email: lwhlbx@163.com

Tel: +86-771-2852965

**A list of the supplementary materials:**

**Figure S1.** Effects of PatA on stress resistance of *M. smegmatis*.

**Figure S2.** Logarithmic phase cultures of BCG/pLJR965, BCG/pLJR965-*patA*_sgRNA_(4 X), BCG/pLJR965-*patA*_sgRNA_(51 X), BCG/pLJR965-*patA*_sgRNA_(110 X), BCG/pLJR965-*patA*_sgRNA_(216 X) strains on 7H10 medium plates with or without 200 ng/mL ATc.

**Figure S3.** Surface morphology of Msm/pMV261, △*patA*/pMV261, △*patA*/pMV261-*patA_msm_,* and △*patA*/pMV261-*patA_mtu_* strains on 7H10 medium plates.


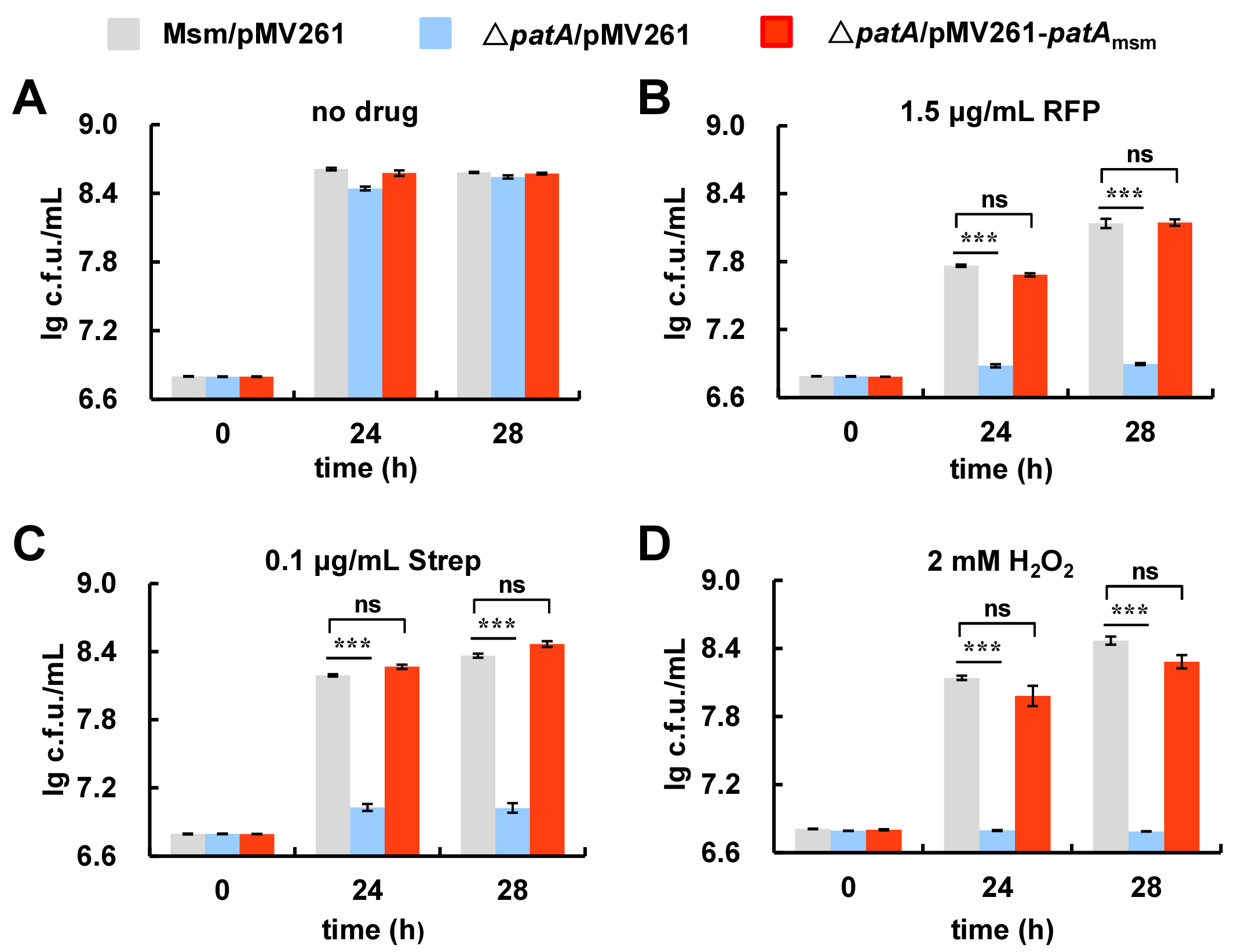


**Fig. S1.** Effects of PatA on stress resistance of *M. smegmatis*. (A)Bacterial counts in Msm/pMV261, △*patA*/pMV261, and △*patA*/pMV261-*patA_msm_* strains grown in 7H9 medium under no drug stress. (B) Bacterial counts in 7H9 medium supplemented with 1.5 mM RFP. (C) Bacterial counts in 7H9 medium supplemented with 0.1 μg/mL Strep. (D)Bacterial counts in 7H9 medium supplemented with 2 mM H_2_O_2_. Three asterisks (***) represents significant difference at the level of *P*<0.001 (two-tailed Student’s t-test) between two groups. Data were expression of mean±SD of six biological replicates.


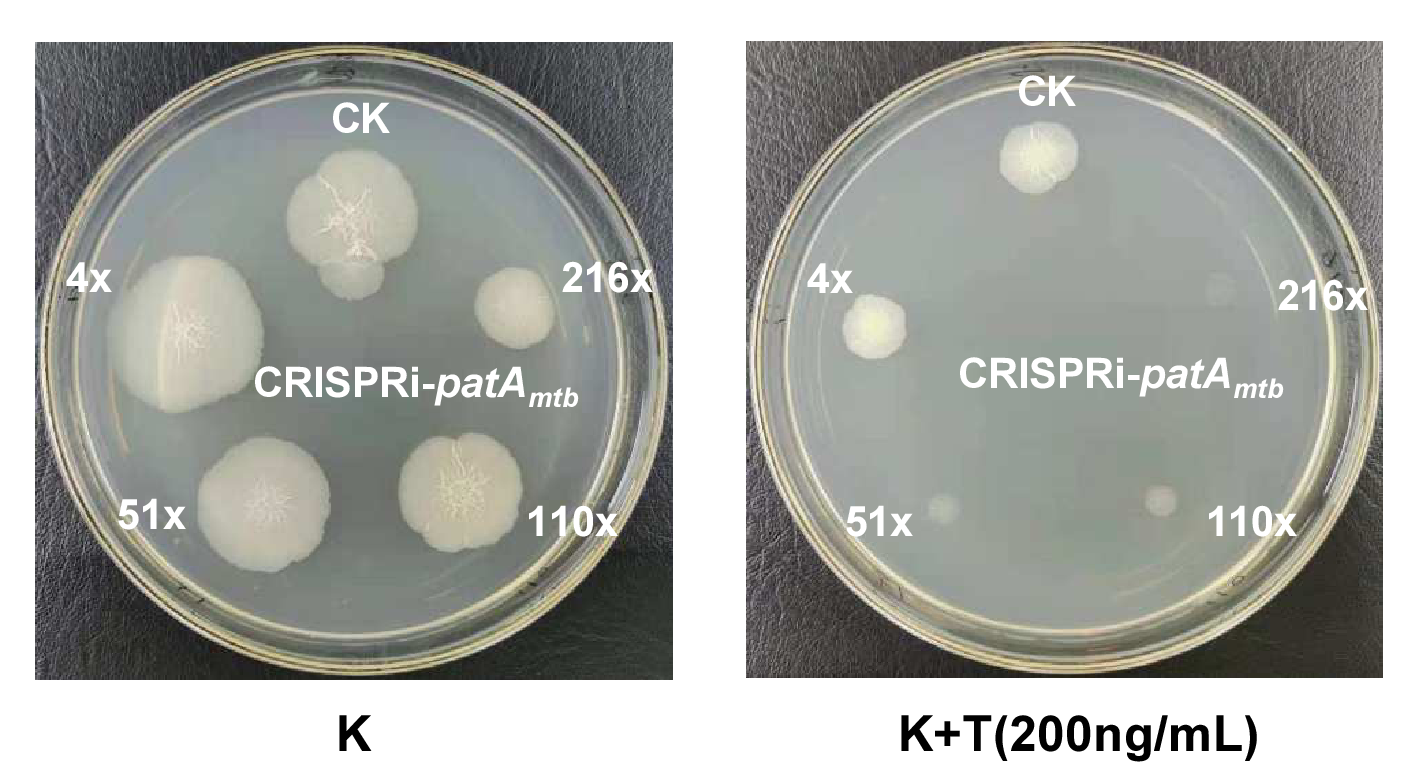


**Fig. S2.** Logarithmic phase cultures of BCG/pLJR965, BCG/pLJR965-*patA*_sgRNA_(4 X), BCG/pLJR965-*patA*_sgRNA_(51 X), BCG/pLJR965-*patA*_sgRNA_(110 X), BCG/pLJR965-*patA*_sgRNA_(216 X) strains on 7H10 medium plates with or without 200 ng/mL ATc.


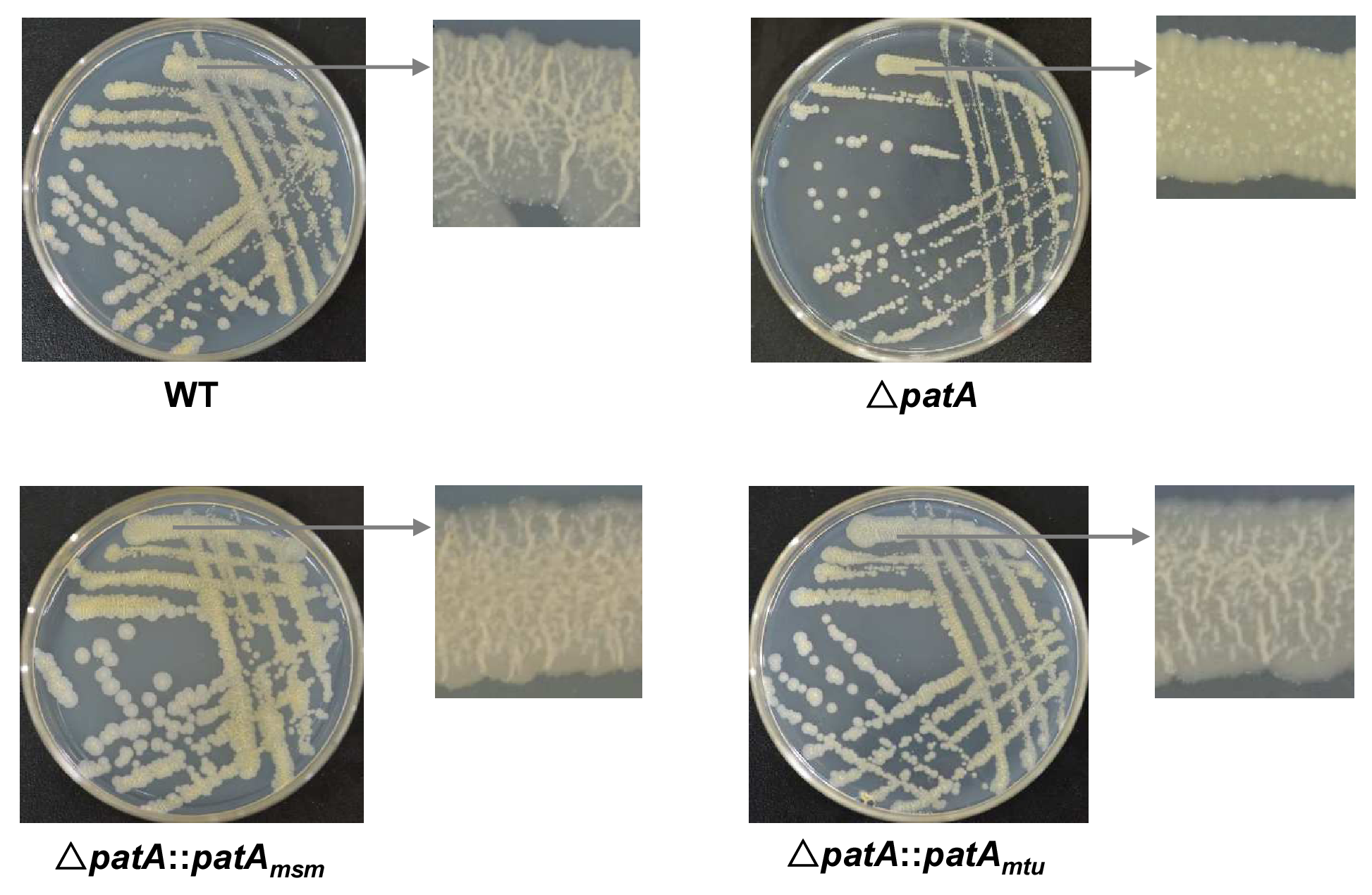


**Fig. S3.** Surface morphology of Msm/pMV261, △*patA*/pMV261, △*patA*/pMV261-*patA_msm_,* and △*patA*/pMV261-*patA_mtu_* strains on 7H10 medium plates.
